# A 41‐Month Case Report: Correction of Anterior Open Bite Using Clear Aligners Combined With Long‐Arm Uprighting Springs

**DOI:** 10.1155/crid/2327408

**Published:** 2026-09-08

**Authors:** Yewei Xin, Yaqiu Chen, Feng Qiu, Chang Liu, Xiaojing Sun, Qian Jiang

**Affiliations:** ^1^ The Department of Orthodontics, Affiliated Stomatology Hospital of Guilin Medical University, Guilin, China; ^2^ Department of Stomatology, Liuzhou Worker′s Hospital, Liuzhou, China; ^3^ The Department of Orthodontics, Stomatology Hospital of Guilin, Guilin, China

**Keywords:** adult orthodontics, anterior open bite, clear aligners, molar mesialization, uprighting spring

## Abstract

Anterior open bite (AOB) is a challenging vertical malocclusion that requires careful control of posterior vertical dimension, and when it is combined with first molar loss, restoration of posterior occlusal support may require either prosthetic replacement or orthodontic molar protraction. This case report describes the treatment of a 22‐year‐old male patient with AOB and compromised first molars who underwent extraction of Teeth 16, 26, and 46 followed by long‐distance molar protraction with clear aligners; similar nonprosthetic approaches have been reported for patients with missing first molars, although clear aligner treatment remains less predictable for complex tooth movements and extraction‐space closure. During space closure, mesial tipping and aligner off‐tracking were managed with long‐arm molar uprighting springs used as auxiliary devices, because molar protraction requires appropriate moment‐to‐force control to reduce uncontrolled crown tipping and improve root paralleling. After 41 months and 82 aligner sets, the extraction spaces were substantially closed, the anterior overbite and overjet were improved, and the facial profile became more harmonious, supporting previous observations that adult AOB correction with aligners may be achievable when vertical control and auxiliary biomechanics are carefully managed. Because root resorption and enamel opacities occurred during treatment, this report also discusses the biological limitations and risk‐control considerations of long‐distance molar protraction in an adult patient, as prolonged orthodontic tooth movement, extraction‐space closure, and aligner or fixed‐appliance therapy may be associated with adverse effects such as external apical root resorption and white spot lesions.

## 1. Introduction

Molar mesialization is frequently used to close extraction or edentulous spaces, adjust the dental midline, and improve posterior occlusal relationships. In patients with missing or structurally compromised first molars, orthodontic space closure may reduce or avoid the need for prosthetic replacement; however, it requires careful biomechanical control, especially when the edentulous span is long.

Predictable molar protraction depends on the amount and quality of alveolar bone, the duration of tooth loss, root morphology, periodontal support, anchorage design, and the clinician′s ability to control tipping, rotation, intrusion, and root parallelism. Space closure should therefore be evaluated not only by crown movement but also by root position, occlusal settling, and periodontal response.

Clear aligners can stage molar movement gradually and offer esthetic and comfort advantages, but complex posterior movements remain less predictable than simple alignment. Since aligner material deformation and anchorage limitations may produce mesial crown tipping or off‐tracking during molar protraction, auxiliary mechanics such as elastics, buttons, attachments, or uprighting springs may be required. This case report presents the use of long‐arm molar uprighting springs as an adjunct to clear aligners for AOB correction combined with long‐distance molar protraction.

## 2. Diagnosis and Etiology

A 22‐year‐old Chinese male presented with a chief complaint of anterior open bite. The patient denied temporomandibular joint (TMJ) symptoms, reported no relevant family or genetic history, and was in good general health.

Initial facial photographs revealed a convex profile with a retruded chin, protrusive upper and lower lips, and an increased lower facial height. The dental midline was not coincident with the facial midline.

Intraoral examination indicated fair oral hygiene and permanent dentition with Tooth 36 missing. The right molars were in a Class I relationship, and the left molars were in a Class II relationship, whereas the canines on both sides exhibited a Class II relationship. A Grade II anterior open bite was observed. Both the maxillary and mandibular arches were ovoid. The maxillary dentition showed mild crowding, whereas the mandibular arch had an edentulous space of approximately 8 mm. The mandibular midline deviated approximately 3 mm to the left. The left posterior segment showed a cusp‐to‐cusp relationship; the right posterior segment was consistent with a Class I intercuspation.

**Figure 1:**
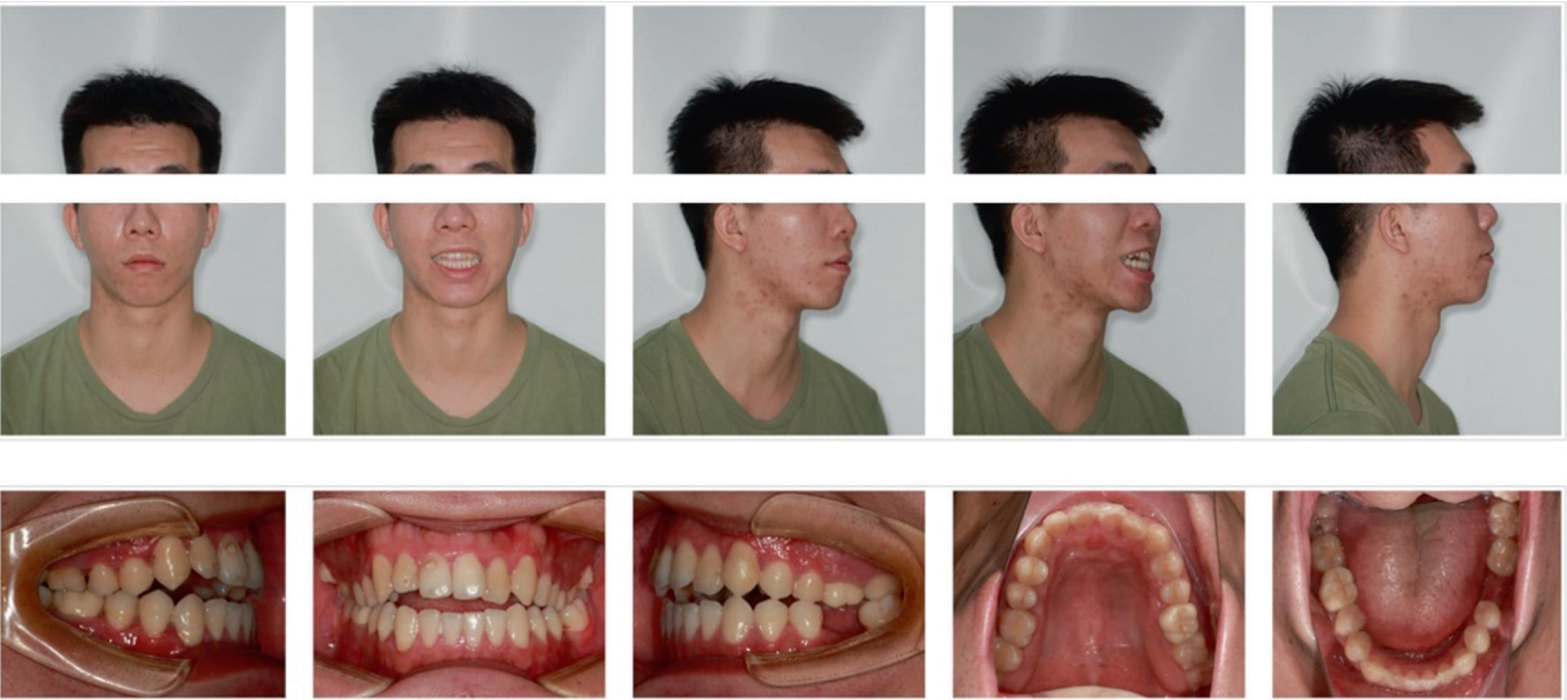
Initial facial and intraoral photographs.

Assessment of the digital models revealed the following: crowding: Grade I maxillary crowding (2 mm), mandibular spacing (8 mm); curve of Spee: 0.8 mm on the left and 1 mm on the right (mean 0.9 mm); overbite and overjet: Grade III anterior open bite (6 mm) and Grade I overjet (2 mm); Bolton ratio: anterior ratio 68.72%, overall ratio 78.99% (Figure 1).

**Figure 2:**
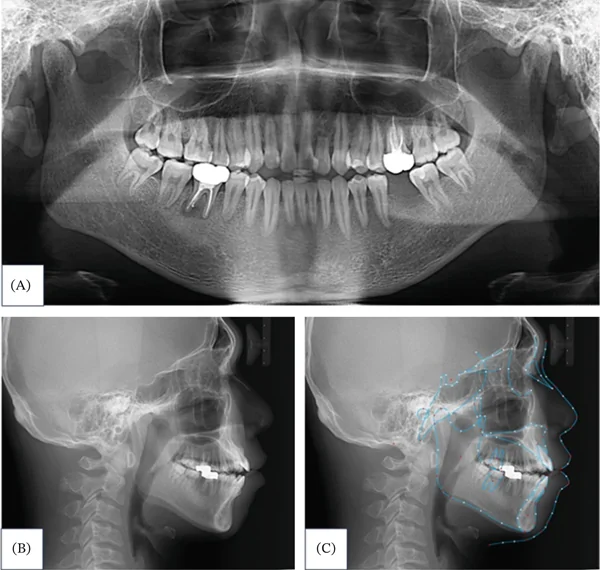
Pretreatment radiographs: (A) panoramic radiograph; (B) lateral cephalogram; and (C) lateral cephalogram tracing.

The panoramic radiograph showed slight asymmetry in the morphology of the bilateral condyles and mild alveolar bone resorption in both jaws. No supernumerary teeth were observed, and Tooth 28 was missing (Figure 2). Cephalometric analysis indicated a skeletal Class I malocclusion (SNA, 81.27°; SNB, 76.35°; ANB, 4.92°) with mandibular retrusion and a steep mandibular plane angle (MP‐SN, 40.05°). The labiolingual inclination of the maxillary and mandibular incisors was within the normal range (U1‐SN, 100.91°; L1‐MP [IMPA], 93.09°). CBCT evaluation demonstrated continuous cortical bone on the bilateral condylar heads, with no obvious pathological change.

### 2.1. Treatment Alternatives

Two treatment alternatives were presented to the patient. Given the patient′s request for esthetics and comfort during the treatment process, both proposed options utilized clear aligner therapy.

**Figure 3:**
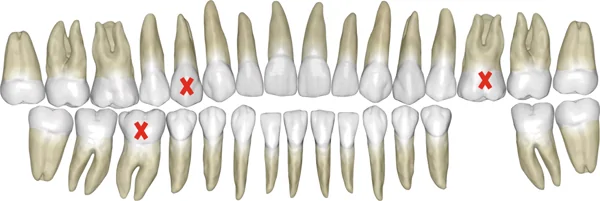
Treatment Plan 1.

Option 1 involved the extraction of Teeth 14, 26, and 46. The core objectives of this plan were to align the dentition and correct the anterior open bite by intruding the posterior segment, thereby inducing a counter‐clockwise rotation of the mandible (Figure 3).

**Figure 4:**
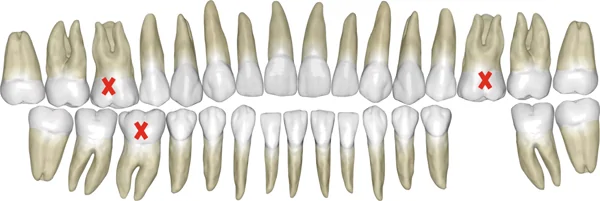
Treatment Plan 2.

Option 2 centered on the extraction of Teeth 16, 26, and 46. Similar to the first option, this plan aimed to align the dentition and correct the open bite through posterior intrusion and the resulting counter‐clockwise rotation of the mandible. However, compared with Option 1, this alternative presented poorer symmetry, and the posttreatment occlusion would not achieve an ideal cusp–fossa relationship (Figure 4).

**Figure 5:**
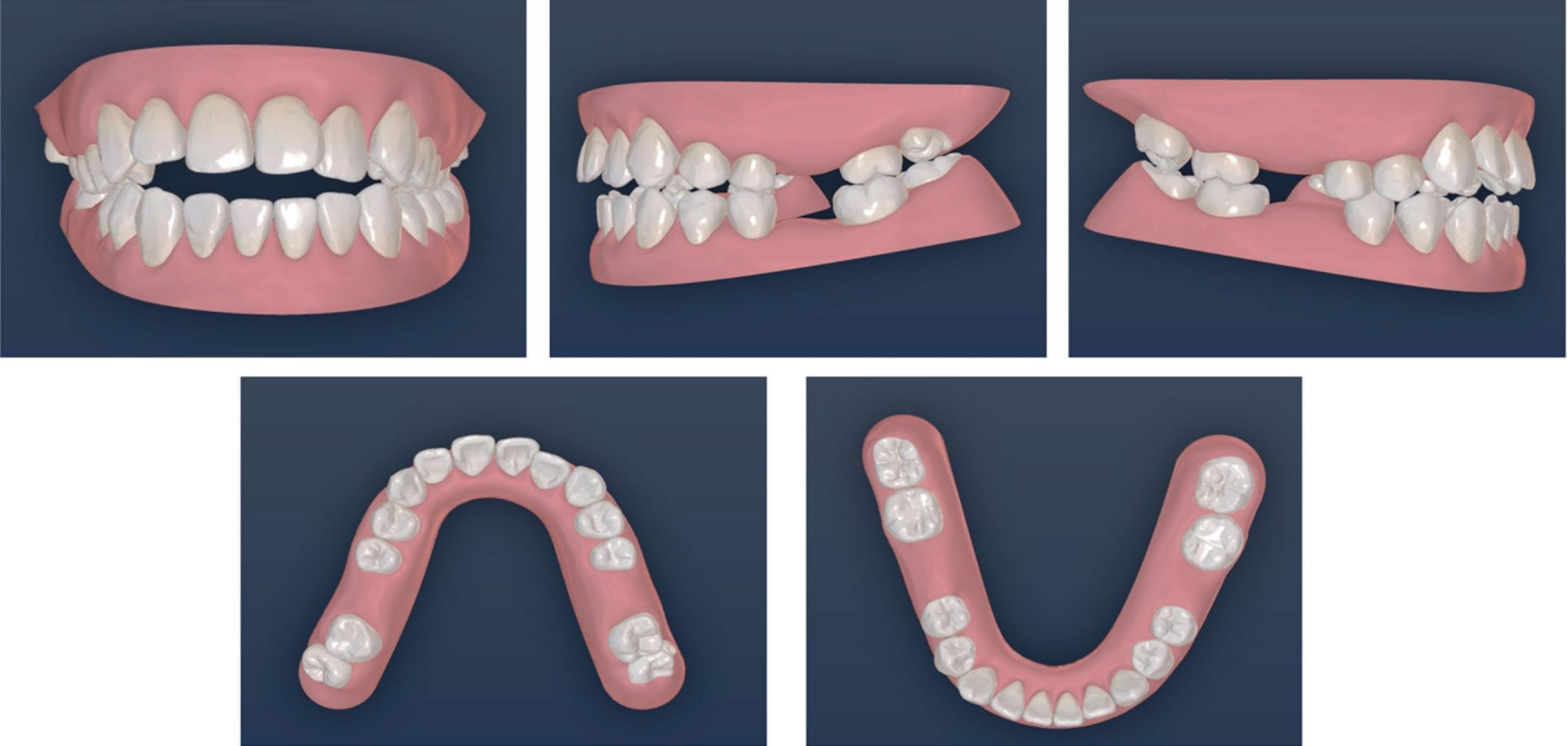
Pretreatment digital model.

**Figure 6:**
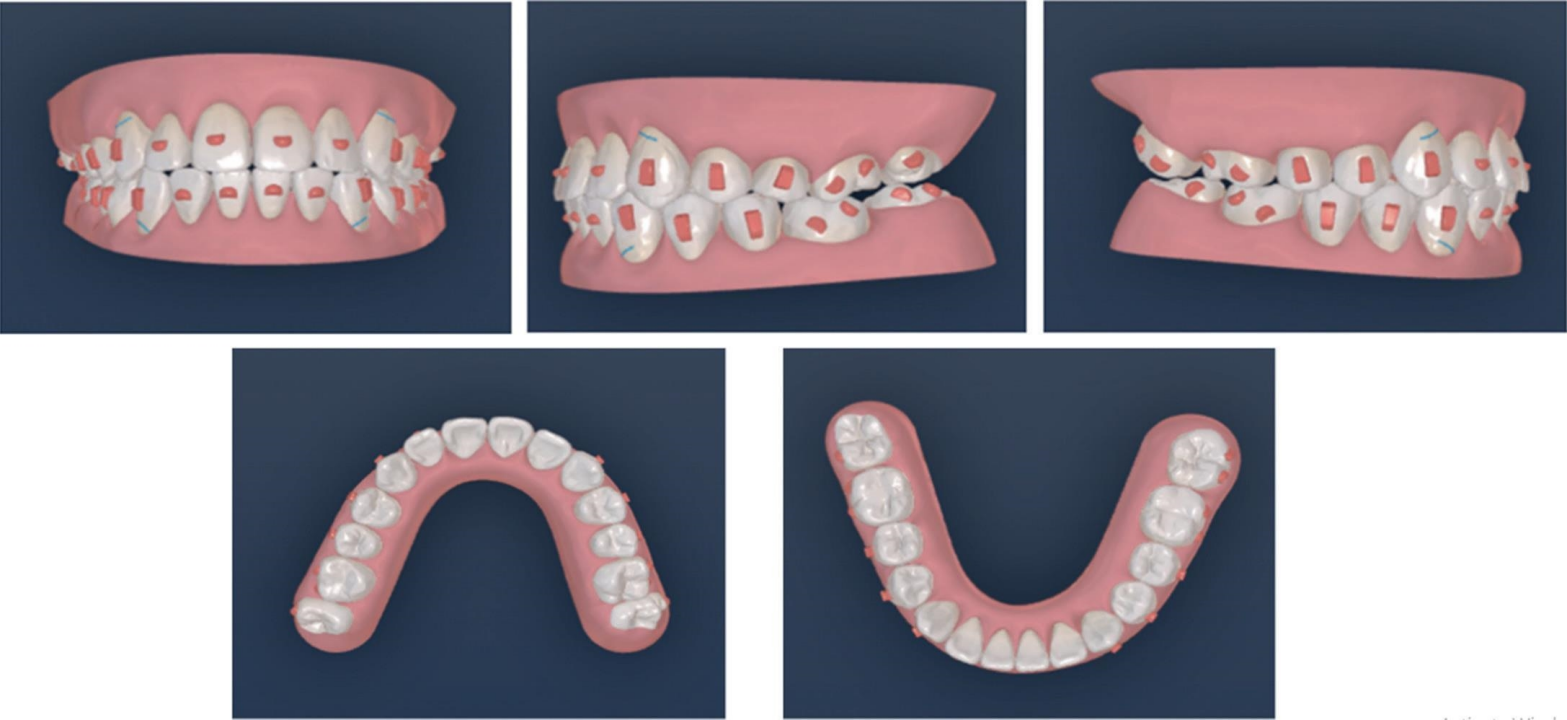
Posttreatment digital model.

Both treatment alternatives aimed to address the closure of the edentulous spaces and the correction of the anterior open bite. The dental spacing was to be resolved through molar protraction, whereas the open bite would be corrected via intrusion of the posterior teeth and retraction of the anterior teeth. Given the patient′s high demands for esthetics and comfort, Option 2 was ultimately selected following thorough consultation and informed consent, and the decision was made to proceed with clear aligner therapy (Figures 5 and 6).

The specific treatment objectives were as follows:

• Close the spaces in the dentition:
• Establish Class I canine and molar relationships:
• Correct the anterior open bite to achieve normal overbite, overjet, and occlusion; improve the facial profile

### 2.2. Treatment Progress

**Figure 7:**
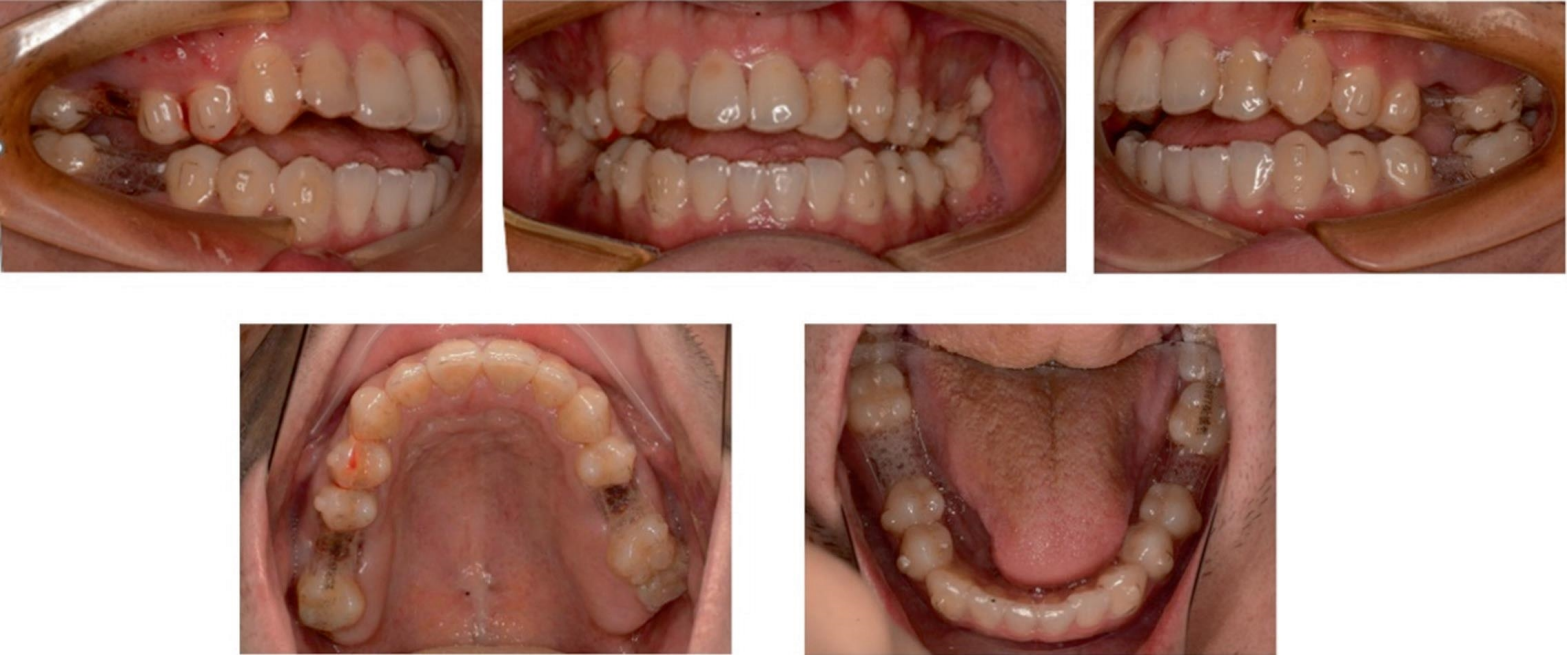
Treatment Stage 1.

Clear aligner therapy was initiated after extraction of Teeth 16, 26, and 46. The patient was instructed to wear each aligner for 14 days and to maintain full‐time wear except during eating, drinking, and oral hygiene procedures (Figure 7). Treatment focused on closure of the extraction spaces and correction of the anterior open bite.

**Figure 8:**
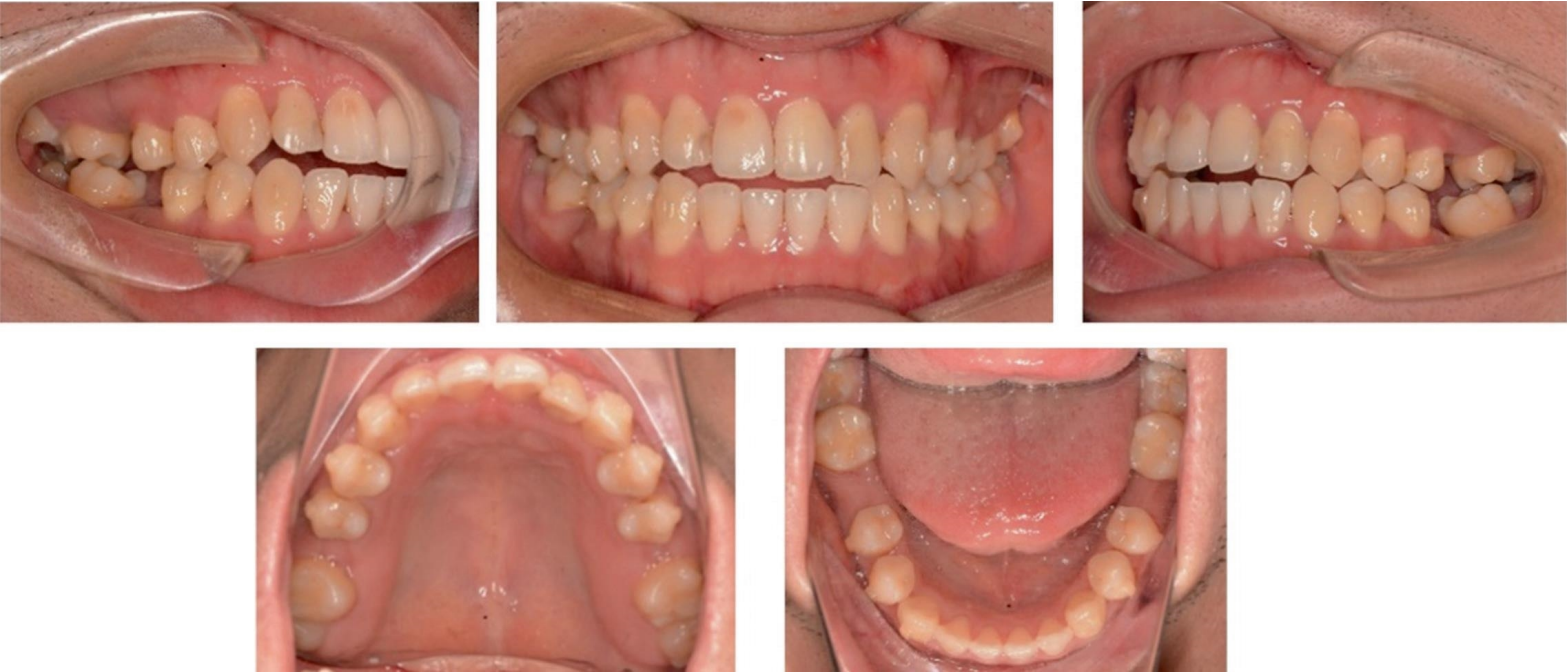
Treatment Stage 2.

At the 8th month of treatment, corresponding to aligner No. 16, the anterior open bite and maxillary anterior crowding had improved (Figure 8). Mesial tipping was observed in the bilateral mandibular posterior teeth. Because aligner tracking remained clinically acceptable at this stage, treatment was continued with close monitoring.

**Figure 9:**
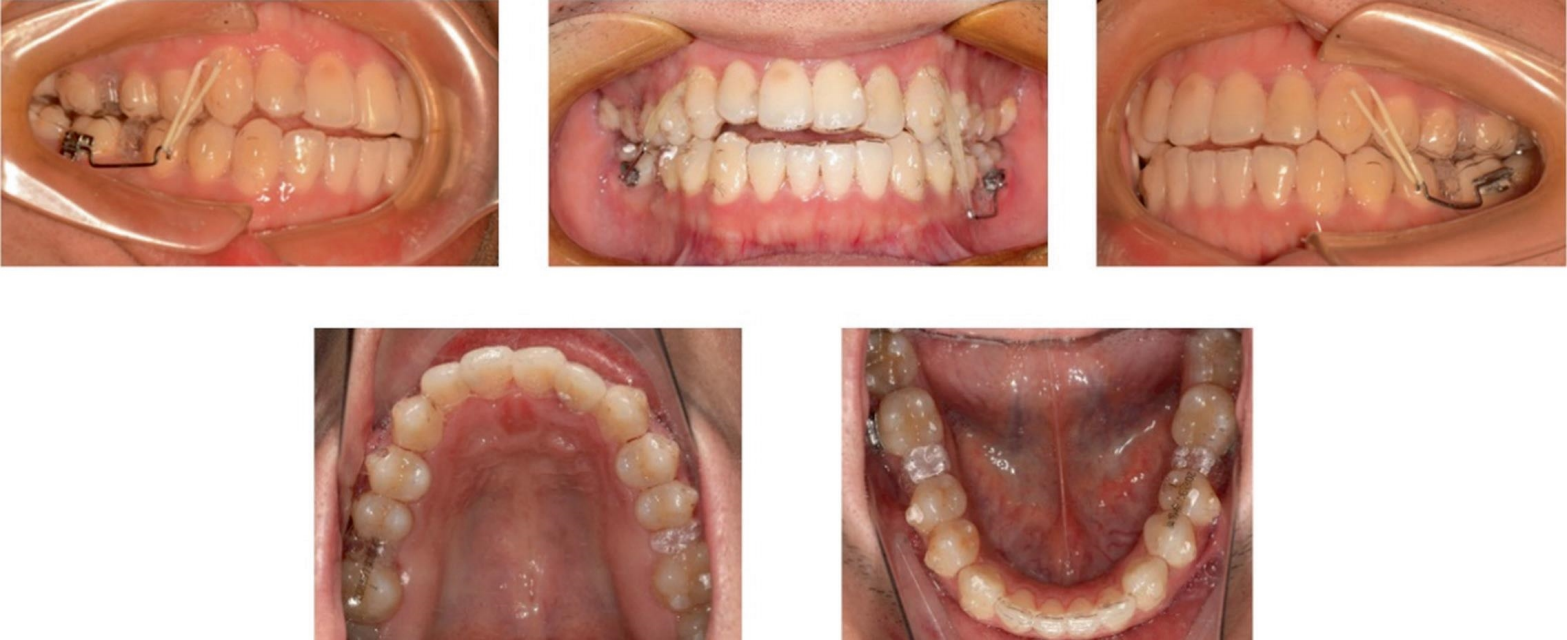
Treatment Stage 3.

At 12 months of treatment (aligner No. 24), aligner off‐tracking occurred due to the mesial tipping tendency of the bilateral mandibular posterior teeth. Consequently, a refinement was initiated. Long‐arm uprighting springs were bonded to the mandibular molars, and cutouts were created on the aligners at the maxillary canines to facilitate the use of Class II elastics. These measures were implemented to retract the maxillary anterior teeth and upright the mandibular molars (Figure 9).

**Figure 10:**
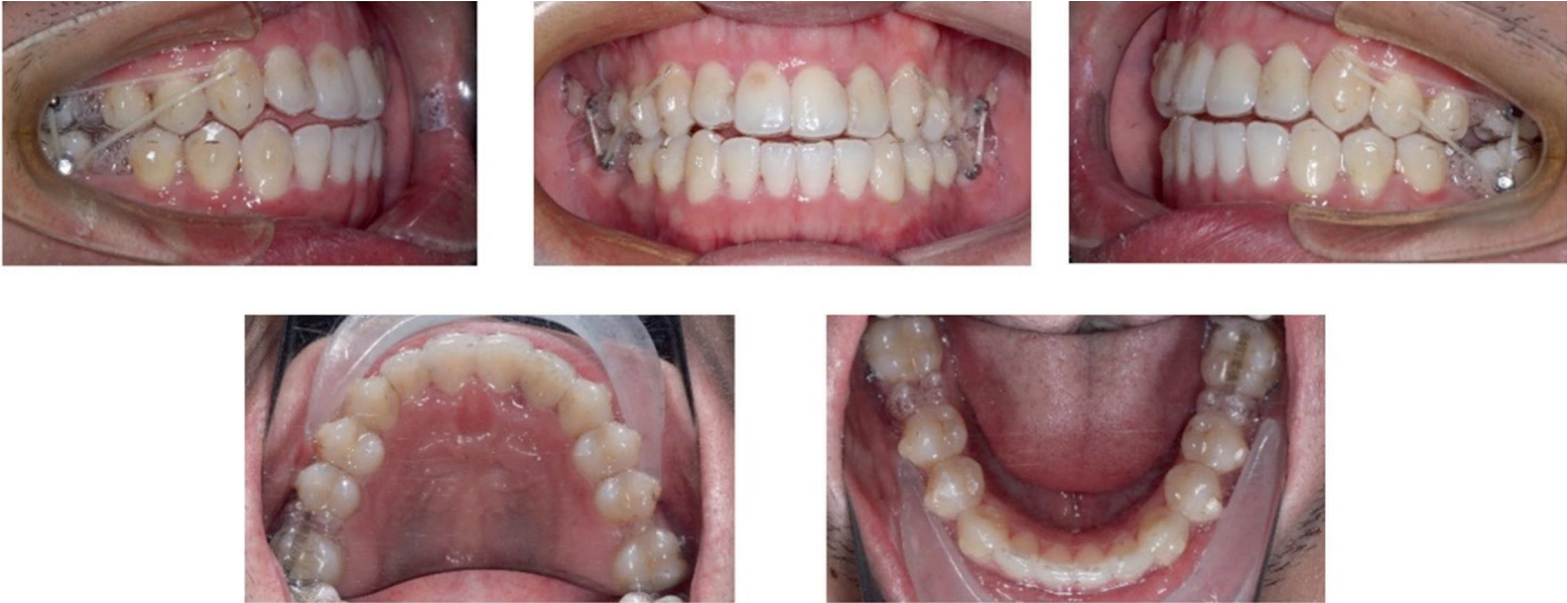
Treatment Stage 4.

At 17 months of treatment, the anterior open bite was resolved, and the tipping of the mandibular right molar had improved. However, at 21 months, aligner off‐tracking occurred due to the patient′s poor compliance with wear time. Consequently, another refinement was initiated. Lingual buttons were bonded, and triangular elastics were employed to close the edentulous spaces (Figure 10).

**Figure 11:**
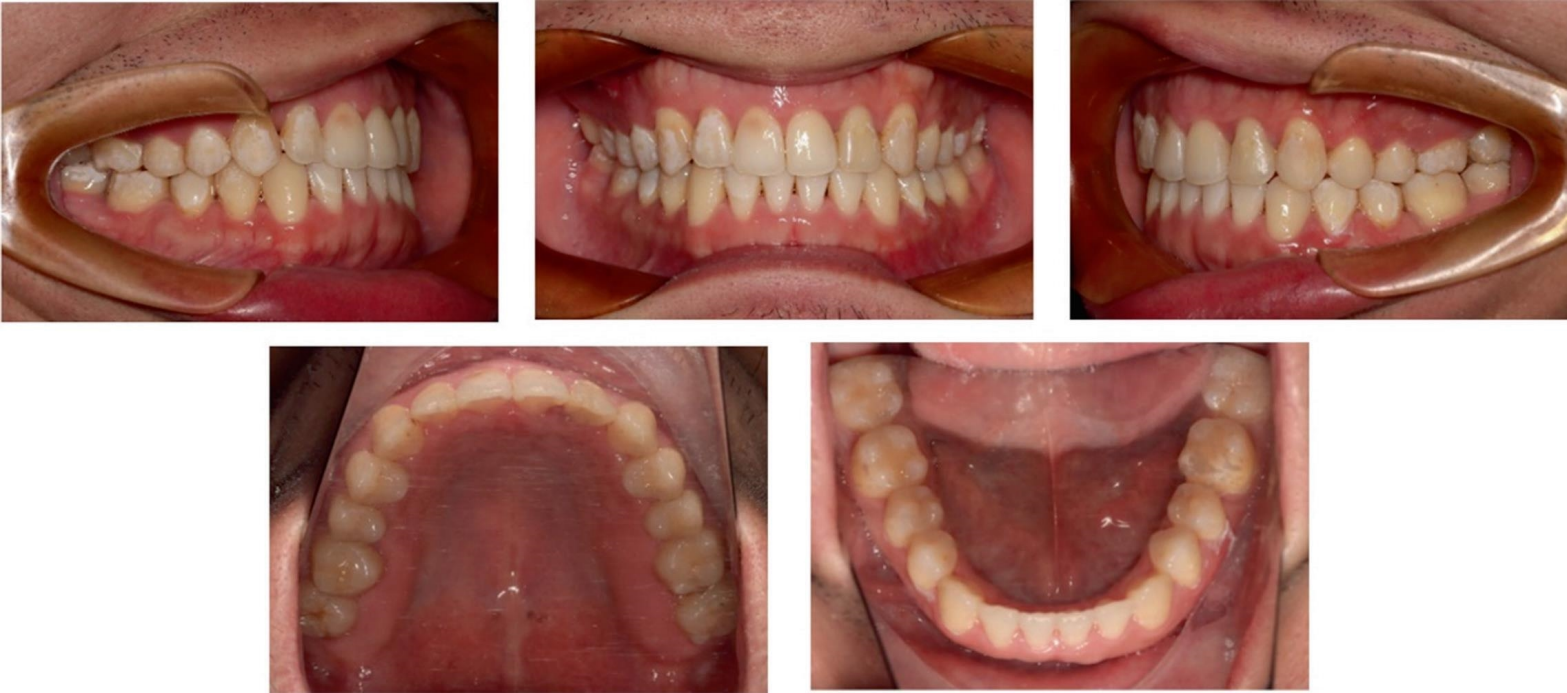
Treatment Stage 5.

At 31 months of treatment, the edentulous spaces were nearly closed, and occlusal detailing was initiated. Active treatment was completed at 41 months. The overall treatment involved 82 aligner sets (Figure 11).

### 2.3. Treatment Results

**Figure 12:**
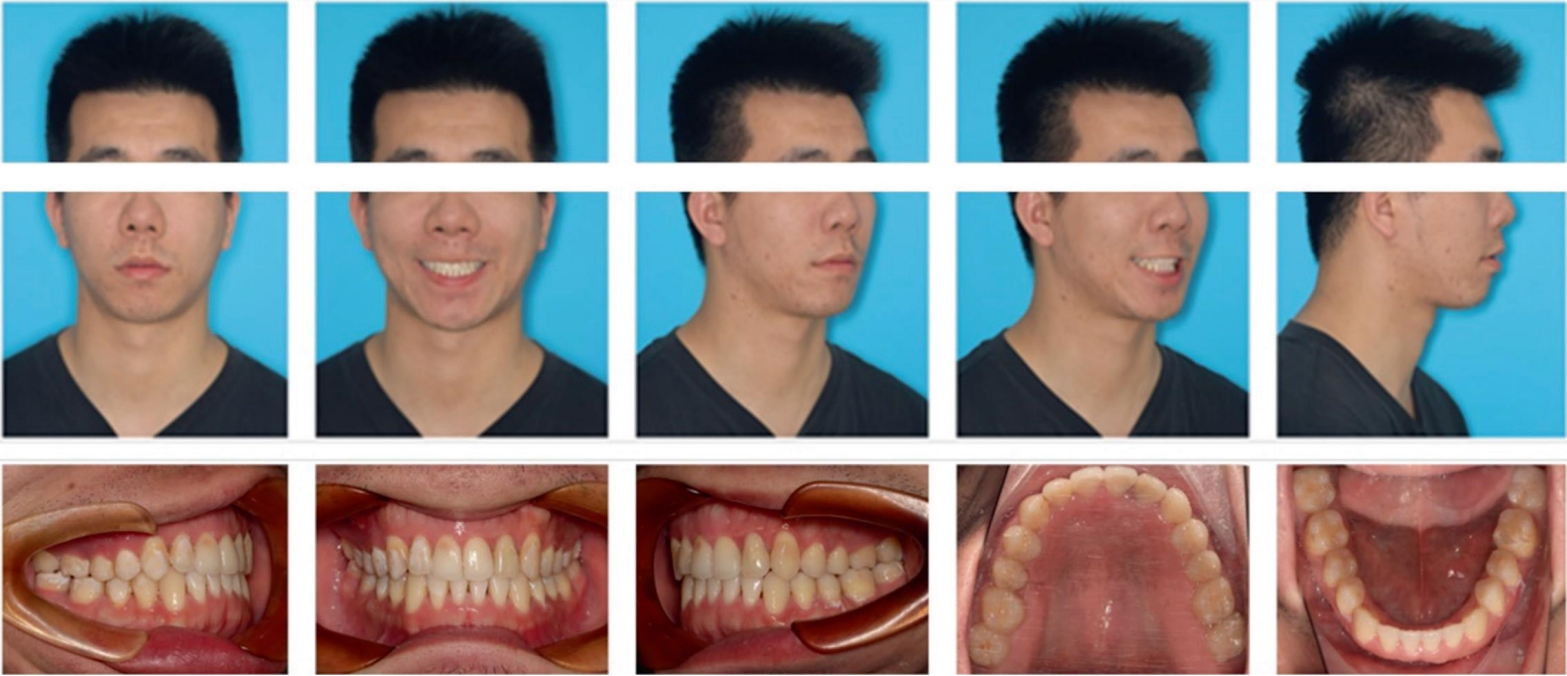
Final facial and intraoral photographs.

**Figure 13:**
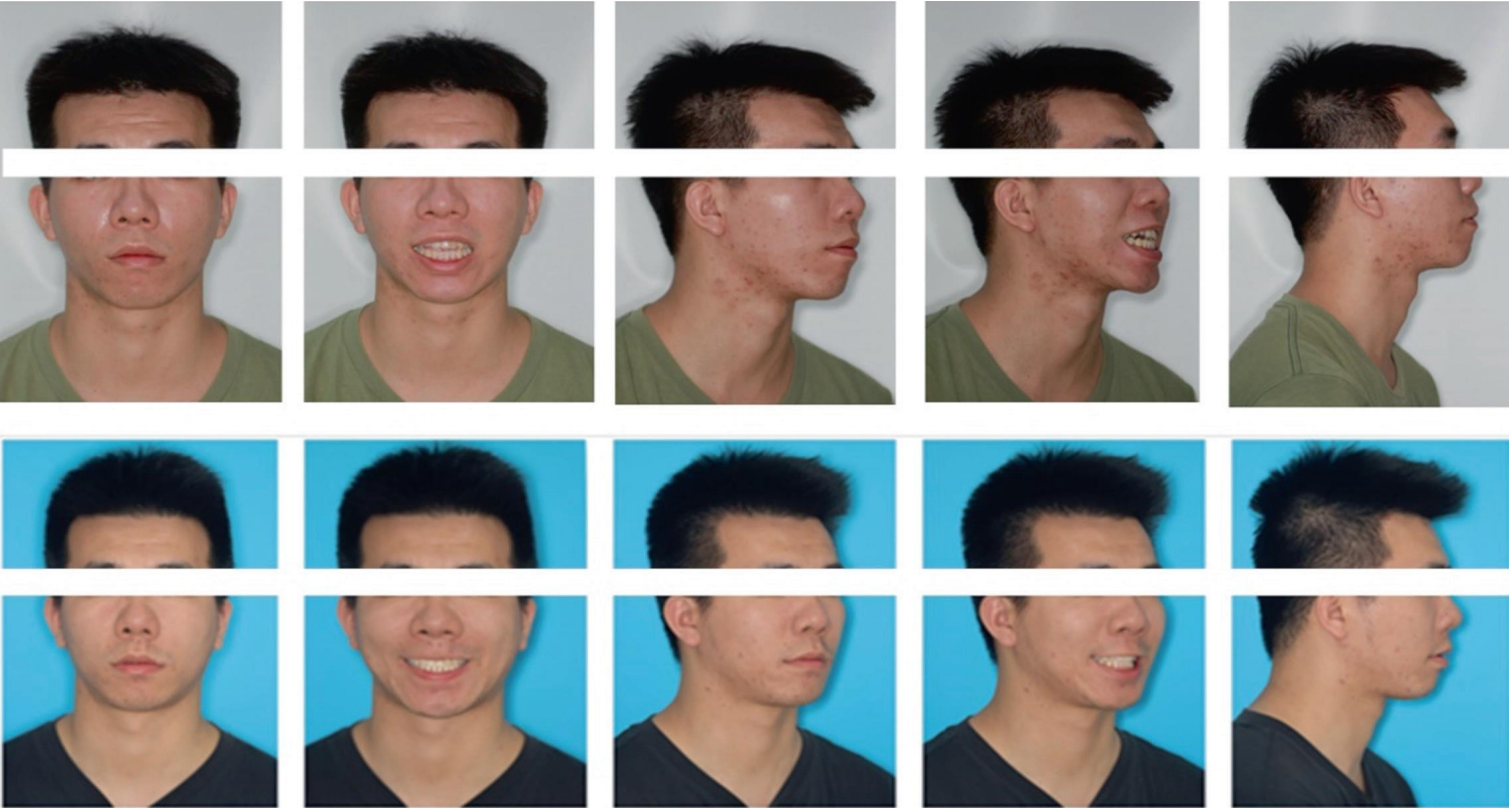
Comparison of appearance: a counterclockwise rotation of the mandible. Upper, pretreatment; lower, posttreatment.

**Figure 14:**
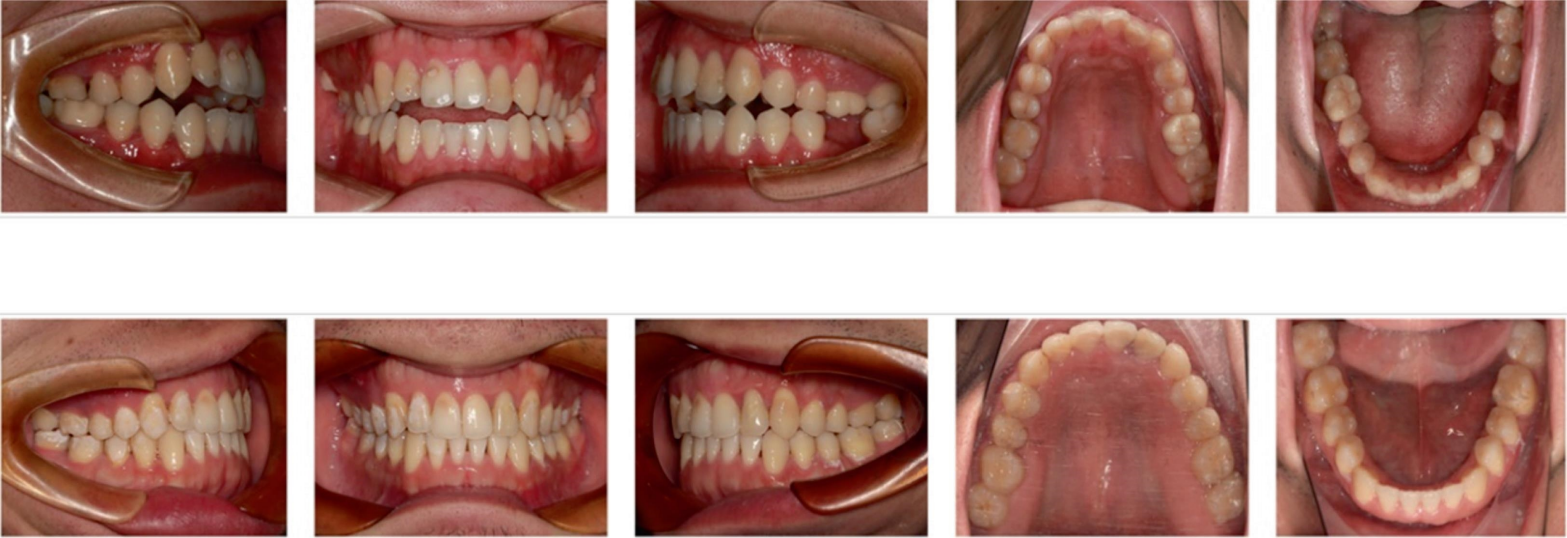
Comparison of intraoral photographs. Upper, pretreatment; lower, posttreatment.

Throughout the treatment period, the clear aligners were changed at 14‐day intervals. After 41 months of active treatment, the maxillary and mandibular dentitions were well aligned. Bilateral Class I canine and molar relationships were achieved, and normal anterior overbite and overjet were established. A slight maxillary‐mandibular dental midline discrepancy remained. The protrusive soft‐tissue profile and anterior open bite improved substantially (Figures 12, 13, and 14).

**Figure 15:**
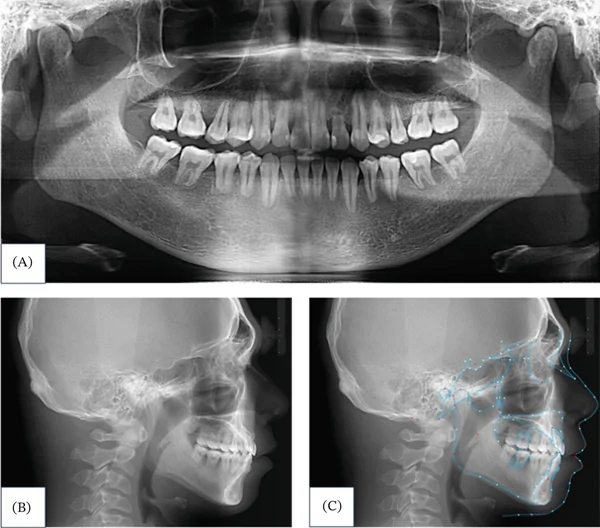
Posttreatment radiographs: (A) panoramic radiograph; (B) lateral cephalogram; and (C) lateral cephalogram tracing.

**Figure 16:**
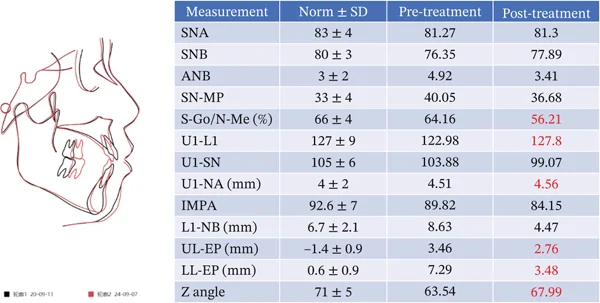
(A) Superimposed cephalometric tracings at pretreatment (black) and posttreatment (red). (B) Cephalometric data comparison before and after treatment.

**Figure 17:**
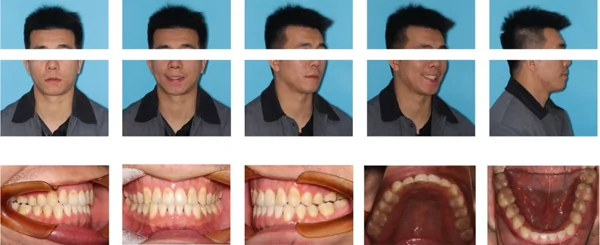
Cephalometric data comparison pretreatment, posttreatment, and 1‐year follow‐up treatment.

**Figure 18:**
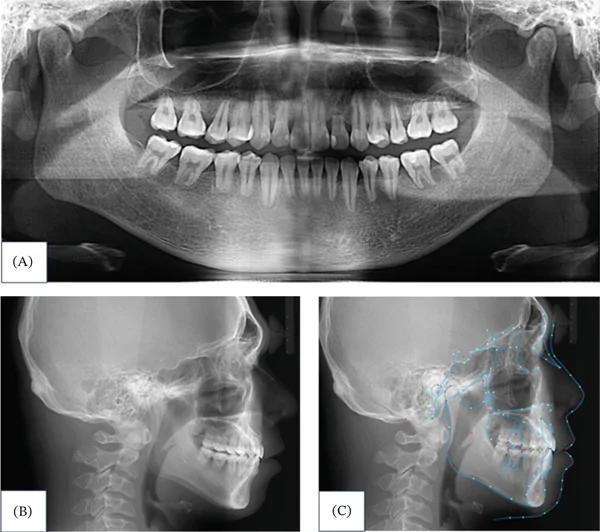
Follow‐up facial and intraoral photographs of the patient 1 year later.

**Figure 19:**
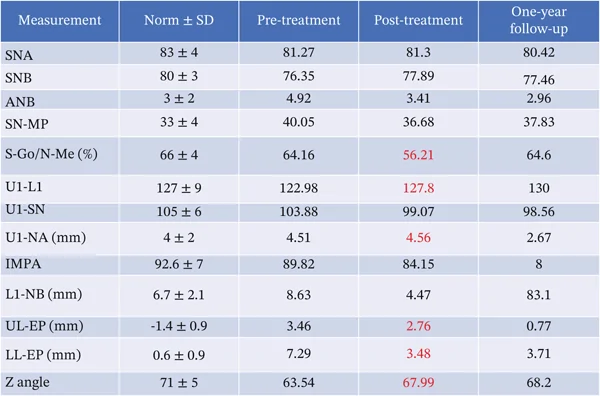
One‐year follow‐up radiographs: (A) panoramic radiograph; (B) lateral cephalogram; and (C) lateral cephalogram tracing.

Posttreatment panoramic radiography indicated acceptable root parallelism and no marked increase in alveolar bone loss compared with pretreatment records (Figure 15). Cephalometric analysis showed changes in sagittal and vertical skeletal measurements, and the values reported in Figures 16 and 17 were cross‐checked for internal consistency. CBCT imaging showed no obvious pathological changes in the bilateral TMJ condyles. Superimposition of pretreatment and posttreatment tracings demonstrated posterior vertical control, anterior tooth retraction, and soft‐tissue profile improvement (Figure 16A). At the 1‐year follow‐up, the occlusion and facial profile were generally maintained. However, posterior intercuspation appeared slightly less complete than immediately after treatment. The 1‐year cephalometric superimposition suggested mild posterior occlusal settling, mainly manifested as slight vertical extrusion of the mandibular molars, without evident loss of overall occlusal stability (Figures 18, 19, and 17). This finding was consistent with the slightly reduced posterior intercuspation observed in the 1‐year intraoral photographs (Figure 18). This minor posterior settling was clinically stable and is being monitored (Figures 18, 19, and 17).

To substantiate the vertical‐correction mechanism and to separate true skeletal change from mesial tipping and the wedge (vertical‐height‐loss) effect, the amounts of posterior‐tooth movement were measured on the superimposed pretreatment and posttreatment lateral cephalometric tracings using a true vertical line (TVL) through nasion as the reference. The maxillary right second molar (17) moved mesially by 4.02 mm and was intruded by 0.25 mm. The maxillary left second molar (27) moved mesially by 2.32 mm and was extruded by 0.29 mm. The mandibular left second molar (37) moved mesially by 3.44 mm and was extruded by 0.56 mm. The mandibular right second molar (47) moved mesially by 5.04 mm and was extruded by 0.47 mm. The SN‐MP angle decreased from 40.05° to 36.68°, a reduction of 3.37°, suggesting counter‐clockwise mandibular rotation. The anterior open bite of 6 mm was corrected to a normal overbite of 2 mm and an overjet of 2 mm.

The cephalometric superimposition (Figure 16) showed that all four second molars underwent controlled mesial movement rather than uncontrolled tipping, supporting the effectiveness of the long‐arm uprighting springs in controlling crown inclination during space closure. Mesialization was greatest for the mandibular right second molar (47, 5.04 mm), followed by the maxillary right second molar (17, 4.02 mm), the mandibular left second molar (37, 3.44 mm), and the maxillary left second molar (27, 2.32 mm). The right‐side molars consistently showed greater mesialization than their left‐side counterparts, possibly reflecting differences in initial extraction‐space size and anchorage conditions; the pre‐existing loss of Tooth 36 may have allowed partial pretreatment drift of 37, accounting for its smaller measured movement.

Vertically, only the maxillary right second molar (17) was intruded (0.25 mm), whereas the remaining three molars exhibited mild extrusion: 27 by 0.29 mm, 47 by 0.47 mm, and 37 by 0.56 mm. This posterior extrusion would, in isolation, increase the wedge effect and rotate the mandible clockwise—opposing open‐bite correction. Nevertheless, the SN‐MP angle decreased by 3.37° (from 40.05° to 36.68°), suggesting that counter‐clockwise mandibular rotation occurred. This finding suggests that the dominant mechanism of open‐bite correction was not posterior intrusion per se, but rather the controlled mesialization of the posterior teeth. By closing the extraction spaces and reducing the anteroposterior dimension of the posterior dental arch, mesialization effectively diminished the wedge and permitted the mandible to rotate counter‐clockwise. The net rotational effect of space closure outweighed the bite‐opening tendency of molar extrusion, resulting in complete correction of the 6‐mm anterior open bite to a normal overbite of 2 mm. The long‐arm uprighting springs played a critical role in this process by converting what could have been uncontrolled mesial tipping into controlled mesial movement, thereby maximizing effective space closure and minimizing the adverse vertical consequences of tipping.

## 3. Discussion

The patient presented with severe anterior open bite and compromised first molars. Extraction of Teeth 16, 26, and 46 was selected to address the existing loss of Tooth 36, maintain a more symmetric extraction pattern, and permit space closure with molar protraction. The intended vertical mechanism was posterior vertical control combined with mandibular counter‐clockwise rotation; however, this mechanism must be interpreted cautiously because molar tipping, posterior occlusal settling, and vertical dimension changes can also influence mandibular‐plane measurements [1, 2].

Clear aligners have been reported as a useful option for adult anterior open bite correction, partly because the occlusal coverage may provide a posterior bite‐block effect and assist vertical control. In this case, however, treatment complexity was increased by long‐distance molar protraction and the absence of temporary anchorage devices [3]. The observed off‐tracking and posterior mesial tipping illustrate the limitations of relying on aligners alone for controlled molar bodily movement [4].

**Figure 20:**
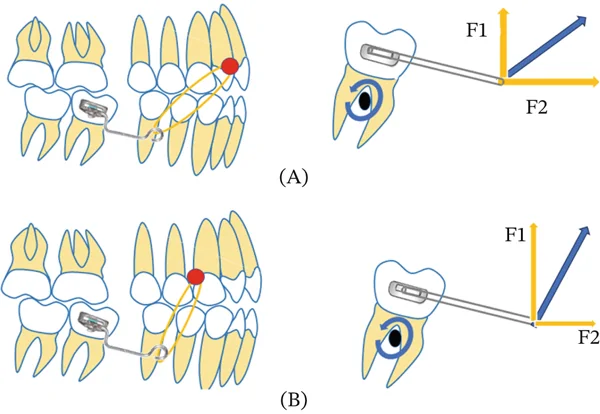
Biomechanical analysis of altering the point of force application on a fixed‐length long‐arm uprighting spring. (A) Point of force application is comparatively farther from the fixed end. (B) Point of force application is comparatively closer with the fixed end.

The auxiliary device used in this case is defined here as a long‐arm molar uprighting spring: a bonded auxiliary arm extending from the molar region that allows elastic traction to be applied at a greater distance from the molar crown, thereby increasing the uprighting moment and modifying the vertical and horizontal force components. Compared with lingual buttons and elastics alone, this design can increase the vertical force component and help reduce uncontrolled mesial crown tipping during molar protraction [5]. The magnitude and direction of the force system may be adjusted by changing the length of the spring arm and the point of force application (Figure 20).

**Figure 21:**
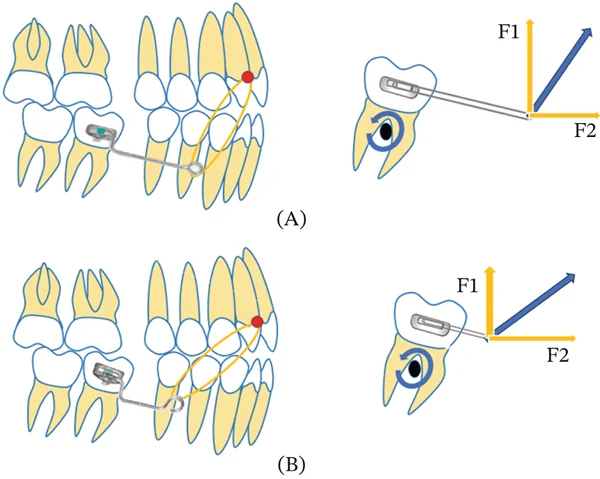
Biomechanical analysis of altering long‐uprighting spring length with a fixed point of force application: (A) with a longer uprighting spring; (B) with a shorter uprighting spring.

Conversely, when the point of traction application is fixed, the length of the spring arm affects the vertical and horizontal force components; a longer arm increases the vertical component and the potential uprighting moment (Figure 21).

This case met most planned clinical objectives, including open‐bite closure, alignment, extraction‐space closure, establishment of acceptable overbite and overjet, and improvement in facial esthetics and oral function. Nevertheless, the result should not be described as an uncomplicated success because clinically relevant biological adverse effects occurred [6].

Several limitations must be emphasized. Root resorption was observed in both anterior and posterior teeth; therefore, it should not be attributed only to teeth subjected to intrusive mechanics [7]. The reasons for this may be multifactorial. First, the correction of the severe anterior open bite was mainly based on posterior vertical control, including posterior intrusion or control of posterior eruption, rather than prolonged intrusion of the anterior teeth [8]. In the posterior region, root resorption may have been associated with long‐distance molar protraction, mesial tipping correction, uprighting mechanics, and posterior vertical control. In the anterior region, possible contributing factors include alignment, torque control, anterior retraction, root morphology, and individual susceptibility. Second, the prolonged treatment duration resulted in sustained mechanical loading on the roots, where the rate of repair may not have kept pace with the rate of damage, further increasing the risk of resorption. Third, preoperative imaging revealed that some teeth had inherent risk factors, such as compromised root morphology, previous endodontic treatment, and periodontal limitations, which may have increased their susceptibility to orthodontically induced root resorption. In addition, the present plan required the mesial movement of three molars over distances exceeding 8 mm in an adult patient; such long‐distance protraction imposes substantial and prolonged demand on alveolar bone and periodontal support, and the observed generalized root resorption and enamel opacities should therefore be interpreted as evidence that the biomechanical load may have approached or exceeded the adaptive capacity of the supporting tissues. This consideration tempers any claim of unqualified success and underscores the need for careful case selection and closer biological monitoring in similar cases.

Extensive enamel opacities were also observed after orthodontic treatment. These changes may be associated with prolonged appliance wear, plaque retention, dietary factors, and inconsistent oral hygiene during a lengthy treatment course [9]. This case therefore highlights the need for strict caries‐risk assessment, repeated oral‐hygiene reinforcement, periodontal maintenance, radiographic monitoring for root resorption, and early treatment modification when biological adverse effects become evident [10].

## 4. Conclusion

This case suggests that clear aligner therapy combined with long‐arm molar uprighting springs may be considered for selected adult patients requiring anterior open‐bite correction and long‐distance molar protraction. The auxiliary spring helped manage mesial tipping during space closure and broadened the biomechanical options available with clear aligners. However, the long treatment duration, root resorption, enamel opacities, and possible loss of posterior intercuspation during follow‐up indicate that this approach requires careful case selection, quantitative monitoring, and transparent discussion of biological risks.

## Author Contributions

**Yewei Xin:** conceptualization, methodology, writing – original draft, writing – review and editing. **Yaqiu Chen:** conceptualization, methodology, investigation, treatment and clinical data acquisition, writing – original draft, writing – review and editing. **Feng Qiu:** investigation, treatment and clinical data acquisition. **Chang Liu:** investigation, treatment and clinical data acquisition. **Xiaojing Sun:** investigation, treatment and clinical data acquisition. **Qian Jiang:** conceptualization, methodology, writing – review and editing, supervision, project administration, funding acquisition.

## Funding

This study was supported by Guangxi Medical and Health Appropriate Technology Development and Application Project (No. S2022153); Guangxi Zhuang Autonomous Region Health Commission Self‐Funded Research Project (No. Z‐C20231971); Project to Improve Basic Research Ability of Young and Middle‐Aged Teachers in Guangxi Colleges and Universities (No. 2024KY0499); and Guilin Science Research and Technology Development Program (No. 20230135‐5‐1).

## Disclosure

All authors have read and approved the final version of the manuscript. Qian Jiang had full access to all of the data in this study and takes complete responsibility for the integrity of the data and the accuracy of the data analysis.

## Ethics Statement

Ethical approval was not required. Written informed consent was obtained from the patient for orthodontic treatment and for publication of anonymized clinical information and images.

## Conflicts of Interest

The authors declare no conflicts of interest.

## Data Availability

The authors confirm that the data supporting the findings of this study are available within the article and/or its supplementary materials. Additional anonymized clinical records are available from the corresponding author upon reasonable request and subject to institutional and patient‐confidentiality restrictions.

## References

[1] Moshiri S. , Araújo E. A. , McCray J. F. , Thiesen G. , and Kim K. B. , Cephalometric Evaluation of Adult Anterior Open Bite Non-Extraction Treatment With Invisalign, Dental Press Journal of Orthodontics. (2017) 22, no. 5, 30–38, 10.1590/2177-6709.22.5.030-038.oar, 29160342. PMC5730134 29160342

[2] Harris K. , Ojima K. , Dan C. , Upadhyay M. , Alshehri A. , Kuo C. L. , Mu J. , Uribe F. , and Nanda R. , Evaluation of Open Bite Closure Using Clear Aligners: A Retrospective Study, Progress in Orthodontics. (2020) 21, no. 1, 10.1186/s40510-020-00325-5, 32830306. PMC7443419 32830306

[3] Zhang H. , Hu H. , Lin D. , Rouzi M. , Shan D. , Lai W. , and Long H. , Clear Aligner Treatment of an Adult Open Bite With Bilateral Missing Mandibular First Molars Through Molar Protraction With Albert Cantilever Arms, International Orthodontics. (2024) 22, no. 4, 100918, 10.1016/j.ortho.2024.100918, 39241603. 39241603

[4] Lyu X. , Cao X. , Chen L. , Liu Y. , Li H. , Hu C. , and Tan J. , Accumulated Biomechanical Effects of Mandibular Molar Mesialization Using Clear Aligners With Auxiliary Devices: An Iterative Finite Element Analysis, Progress in Orthodontics. (2023) 24, no. 1, 10.1186/s40510-023-00462-7, 37032410. PMC10083150 37032410

[5] Becker K. , Wilmes B. , Grandjean C. , Vasudavan S. , and Drescher D. , Skeletally Anchored Mesialization of Molars Using Digitized Casts and Two Surface-Matching Approaches : Analysis of Treatment Effects, Journal of Orofacial Orthopedics/Fortschritte der Kieferorthopädie. (2018) 79, no. 1, 11–18, 10.1007/s00056-017-0108-y, 29134232. 29134232

[6] Weltman B. , Vig K. W. , Fields H. W. , Shanker S. , and Kaizar E. E. , Root Resorption Associated With Orthodontic Tooth Movement: A Systematic Review, American Journal of Orthodontics and Dentofacial Orthopedics. (2010) 137, no. 4, 462–476, 10.1016/j.ajodo.2009.06.021. 20362905

[7] Yassir Y. A. , McIntyre G. T. , and Bearn D. R. , Orthodontic Treatment and Root Resorption: An Overview of Systematic Reviews, European Journal of Orthodontics. (2021) 43, no. 4, 442–456, 10.1093/ejo/cjaa058, 33215186. 33215186

[8] Kim S. J. , Sung E. H. , Kim J. W. , Baik H. S. , and Lee K. J. , Mandibular Molar Protraction as an Alternative Treatment for Edentulous Spaces: Focus on Changes in Root Length and Alveolar Bone Height, Journal of the American Dental Association. (2015) 146, no. 11, 820–829, 10.1016/j.adaj.2015.04.025, 26514887. 26514887

[9] Raghavan S. , Abu Alhaija E. S. , Duggal M. S. , Narasimhan S. , and Al-Maweri S. A. , White Spot Lesions, Plaque Accumulation and Salivary Caries-Associated Bacteria in Clear Aligners Compared to Fixed Orthodontic Treatment. A Systematic Review and meta- analysis, BMC Oral Health. (2023) 23, no. 1, 599, 10.1186/s12903-023-03257-8. 37635207 PMC10463770

[10] Yazarloo S. , Arab S. , Mirhashemi A. H. , and Gholamrezayi E. , Systematic Review of Preventive and Treatment Measures Regarding Orthodontically Induced White Spot Lesions, Dental And Medical Problems. (2023) 60, no. 3, 527–535, 10.17219/dmp/140964, 37815515. 37815515

